# A Transperineal Biopsy of the Prostate Does Not Require Routine Antibiotic Cover

**DOI:** 10.7759/cureus.61552

**Published:** 2024-06-02

**Authors:** David Dryhurst, Abdullatif Aydin, Nkwam Nkwam

**Affiliations:** 1 Urology, Princess Royal University Hospital, King's College Hospital NHS Foundation Trust, London, GBR; 2 Urology, Guy's Hospital, King's College London, London, GBR; 3 Urology, King's College Hospital NHS Foundation Trust, London, GBR

**Keywords:** antibiotic stewardship program, amr, local anaesthetic, antibiotic, transrectal prostate biopsy, prostate cancer, sepsis, infection, transperineal prostate biopsy

## Abstract

Introduction

A transperineal ultrasound-guided prostate biopsy (TPB) under local anaesthetics (LA) after a prostate MRI scan is the gold standard for performing a prostate biopsy in patients with suspected prostate cancer. It has superseded transrectal ultrasound-guided prostate biopsy (TRUSB). Historically, TRUSB by definition was performed in a contaminated environment and was routinely covered with antibiotics to reduce the risks of infection. Despite this, the rate of post-biopsy urosepsis has been documented to be as high as 5% in some series. In the transition from TRUSB to the establishment of a TPB under LA service in our unit, we continued to use a single dose of oral antibiotics for all patients attending for biopsy. The aim of this study is to establish whether the use of single-dose antibiotics has any effect on morbidity rates post-TPB.

Methods

A retrospective analysis of complications was carried out on 326 consecutive patients, who underwent TPB over a six-month period. One cohort of patients were biopsied with no antibiotic cover (n=149, 45.7%) as compared to another cohort who were given a single dose of oral antibiotics (n=177, 54.3%). Those patients in the group receiving antibiotics received either a single dose of co-amoxiclav or a single dose of ciprofloxacin. Patients with indwelling urethral catheters or with a urinary tract infection (UTI) were excluded from the analyses. All patients were followed- up after a multidisciplinary team meeting discussion (MDT) with either a telephone or a face-to-face consultation.

Results

A total of 324 (99.4%) patients did not report post-procedural complications. Two patients from the antibiotic group presented with infectious complications (1.1%); one patient was admitted with a prostate abscess and required drainage under general anaesthesia, and another was admitted with urosepsis requiring intravenous antibiotics. In the group who did not receive antibiotics, there were no complications reported, which was not significantly different compared to the antibiotic group (p=0.50).

Conclusion

Our results demonstrate that the routine use of single-dose antibiotics with TPB does not affect morbidity rates. On the basis of this investigation, we have now stopped using routine antibiotic cover for patients undergoing an LA TPB.

## Introduction

Prostate cancer (PCa) is the most frequently diagnosed cancer among men in more than half of the countries in the world, with an estimated 1.4 million new cases in 2020. The highest age-standardized incidence rates are seen in Northern and Western Europe, the Caribbean, Australia/New Zealand, North and South America, and Southern Africa.

It is the leading cause of cancer death among men in a quarter of the world’s countries, with an estimated 375,000 deaths in 2020, and it is the most common cancer in men in the United Kingdom (UK), with 40,300 cases diagnosed in 2015 [1].

Risk factors include age, ethnicity, and family history of the disease., There is evidence that circulating insulin-like growth factor-I (IGF-I), which is influenced by environmental factors, is related to higher PCa risk [2]. Obesity is associated with a higher incidence of aggressive PCa [3].

Global differences in PCa incidence and mortality can be attributed to differences in screening, imaging, access to care, and healthcare infrastructure [4-6].

PCa can present with an elevated prostate-specific antigen (PSA) blood test based on presymptomatic patient testing, with lower urinary tract symptoms (LUTS) or with symptoms related to metastatic diseases, such as back pain or pathological fractures.

The majority of patients in the UK are diagnosed following investigations for an elevated PSA. When diagnosed and treated at localized stages, PCa has a 97% 5-year cancer-specific survival compared with 30% in the metastatic setting [4].

A transperineal ultrasound-guided prostate biopsy (TPB) has a better pickup rate of anteriorly located cancers compared to a transrectal ultrasound-guided prostate biopsy (TRUSB) [7]. An elevated PSA does not necessarily require further investigations in older, comorbid patients with limited life expectancy and no evidence of metastatic disease.

The histological grading of PCa is based on the modified Gleason grading and scoring system [8], which, together with the serum PSA level and the staging investigations (MRI, CT, bone scan, and prostate-specific membrane antigen PET (PSMA PET)), enables the stratification of PCa into risk groups. Individual treatment is then offered based on risk stratification.

Histological diagnosis requires tissue usually obtained by a prostate biopsy. Historically, this was performed by TRUSB, originally performed ‘blind’ (i.e. digitally guided) evolving onto ultrasound-guided. A further evolution in biopsy and now the gold standard is TPB.

Like TRUSB, TPB can be associated with a number of complications, including sepsis, haematuria, haemospermia, urinary tract infections (UTIs), prostatitis, urinary retention, and epididymo-orchitis [9]. Of these, sepsis is the most serious complication.

Historically, TRUSB had sepsis rates of up to 5% [10]. TPB offers a lower rate of infection [10], and like TRUSB, it can be performed under local anaesthetics (LA) in an office-based setting. Additionally, TPB has the advantage of an improved diagnostic sensitivity for clinically significant PCa [7].

TRUSB was essentially a transfaecal route of biopsy, and as such, it was routinely covered by the administration of antibiotics in order to lower the risk of sepsis. TPB is transcutaneous, avoiding faecal contamination, and hence it has a lower risk of infection.

## Materials and methods

In our unit, we perform TPB under LA for over 95% of men undergoing TPB. Those unable to undergo TPB under LA underwent the procedure under sedation or general anaesthesia in our day surgery unit.

We undertook a retrospective analysis of 326 patients attending TPB under LA over a six-month period between January 2023 and July 2023. One clinical team routinely covered their biopsy list with antibiotics whereas the other team did not use antibiotics. The indication for TPB was based on an elevated PSA, abnormal digital rectal examination, abnormal prostatic MRI (mpMRI), or as part of active surveillance for pre-existing adenocarcinoma of the prostate. It was not blinded or placebo-controlled.

Patients were allocated to the lists by administrative staff who were unaware as to the practices of antibiotic cover by each consultant and his team.

At the time of the biopsy appointment, patients attending for the biopsy had their notes, results, observations, and urinalysis checked. Patients with indwelling urethral catheters or with a UTI were excluded from the analyses.

One cohort were biopsied with no antibiotic cover (n=149, 45.7%) as compared to another cohort who were given a single dose of oral antibiotics (n=177, 54.3%). Those patients in the group receiving antibiotics were given either a single dose of 625 mg co-amoxiclav or, in the case of penicillin allergy, a single dose of 500 mg ciprofloxacin.

Following skin prep with chlorhexidine, both groups underwent biopsies under LA using ethyl chloride spray to numb the perineum and then 20 mL of 1% lidocaine LA infiltration. Following infiltration with LA, a five-minute interval was timed prior to starting biopsies. Biopsies were then taken using the PrecisionPoint® Transperineal Access System (PPTAS; Perineologic, Cumberland, MD) [11].

Statistical analysis was performed using Fisher’s exact test in GraphPad Prism software (GraphPad Software, San Diego, CA). This analysis was performed as part of routine clinical governance, and ethics committee approval was not required.

## Results

Following discussion at our multidisciplinary team meeting (MDT), patients were followed up in the clinic with either a telephone or face-to-face consultation and asked about any infectious complications following their biopsy. No patients were lost to follow-up.

A total of 324 (99.4%) of the 326 patients did not report any infectious post-procedural complications. Two patients of the 177 (1.1%) from the antibiotic group reported complications; one patient was admitted with urosepsis and a prostate abscess 10 days post procedure. This was approximately 2 cm in diameter and required drainage under general anaesthesia. Escherichia coli was grown on blood and urine cultures. He subsequently went on to make a full recovery. In the same group, another patient was admitted with urosepsis seven days post procedure. He was treated with intravenous amikacin and co-amoxiclav for 48 hours as an inpatient and discharged on oral antibiotics. Blood and urine cultures were negative. He also went on to make a full recovery.

Both of the above patients had no pre-existing comorbidities and had performance status 0. In the group that did not receive antibiotics, there were no complications reported (0/149; p=0.50), which was not significantly different when compared to the antibiotic use group (Table 1).

**Table 1:** Statistical analysis Fisher's exact test The two-tailed p value equals 0.5022. The association between rows (groups) and columns (outcomes) is considered to be not statistically significant.

	Outcome 1 infection	Outcome 2 no infection	Total
Group 1 No antibiotic	0	149	149
Group 2 Antibiotic	2	175	177
Total	2	324	326

## Discussion

Bacterial antimicrobial resistance (AMR) occurs when drugs used to treat infection become less effective, and it has become a major concern for clinicians, with an estimated 4.95 million deaths in 2019 associated with AMR [12]. TPB has already been demonstrated to have a very low risk of infection in comparison to TRUSB.

A systematic review [13] of eight studies involving 2,368 patients from the United States of America, Europe, and the UK who underwent TP biopsies using antibiotic prophylaxis (AP) versus 1,294 patients who underwent TPB without AP was performed. This analysis reported pooled rates of post-procedure sepsis of 0.13% in the antibiotics group vs 0.09% in the no-antibiotics group (RR: 1.09, 95% CI: 0.21-5.61, p = 0.92). This systematic review found no significant difference in infection rate, fever, sepsis, or readmission rates after TPB between those cases utilizing AP and those cases without AP.

Our study, involving an urban, ethnically diverse UK patient population has demonstrated no significant difference between patients receiving AP and those not receiving AP. 

The age range was 45-85, with a median age of 67 and a mean of 65.94 years. The total number of patients was 326. Ethnicity was 183 (56.1%) White, 63 (19.3%) Black, 17 (5.2%) Asian, and 63 (19.3%) not stated on the patient records (Table 2).

**Table 2:** Patient demographics

Ethnicity	Age in years	Number and percentage of total
White	Mean 63.5, Median 64 (range 50-85)	183 (56.1%)
Black	Mean 64, Median 64 (range 45-80)	63 (19.3%)
Asian	Mean 64.7, Median 63 (range 55-79)	17 (5.2%)
Not stated	Mean 63.47, Median 64 (range 50-76)	63 (19.3%)

In total, 284 (87%) of the biopsied patients were found to have prostate adenocarcinoma, and 42 (13%) of the biopsied patients had no cancer.

In the move from TRUSB to TPB, our routine practice had been to continue to offer AP for the procedure. In keeping with other work [13], we have demonstrated that this did not improve the outcome, and as such, we no longer offer this as routine management.

Given the global antibiotic resistance crisis and the need for the adoption of antibiotic stewardship programmes [14], a reduction in routine AP, when proven unnecessary, is a positive move. The omission of routine AP not only reduces the risk of AMR but also reduces the risk of significant side effects associated with antibiotics such as fluoroquinolones, including tendonitis, aortitis, agitation, reduction in blood glucose levels, and aortic dissection, co-amoxiclav rash, Clostridium difficile diarrhoea, and undiagnosed penicillin allergy. Although it must be acknowledged that a single dose of antibiotic is unlikely to be associated with most of these potential side effects, from a patient perspective, the avoidance of potentially serious medication side effects is laudable [15].

The avoidance of routine AP in TPB has significant advantages from an antibiotic stewardship perspective, reducing the risk of AMR and avoiding unnecessary antibiotic-related complications in the patient. Additionally, it reduces the costs associated with performing TPB of the prostate.

## Conclusions

Our results demonstrate that performing TPB in patients without antibiotic cover does not increase the risk of infection. There was no statistical significance demonstrated between the two groups. Our study is in keeping with others that have demonstrated no significant difference in cases of sepsis in patients undergoing TPB without antibiotic cover.

On the basis of this work, we have now stopped using routine antibiotic cover for patients undergoing LA TPB in our department.
